# Angiotensin System Modulations in Spontaneously Hypertensive Rats and Consequences on Erythrocyte Properties; Action of MLN-4760 and Zofenopril

**DOI:** 10.3390/biomedicines9121902

**Published:** 2021-12-14

**Authors:** Tomas Jasenovec, Dominika Radosinska, Marta Kollarova, Peter Balis, Ezgi Dayar, Iveta Bernatova, Stefan Zorad, Norbert Vrbjar, Sona Cacanyova, Jana Radosinska

**Affiliations:** 1 Faculty of Medicine, Institute of Physiology, Comenius University in Bratislava, Sasinkova 2, 813 72 Bratislava, Slovakia; tomas.jasenovec@fmed.uniba.sk (T.J.); marta.husseinova@fmed.uniba.sk (M.K.); 2 Faculty of Medicine, Institute of Immunology, Comenius University in Bratislava, Odborarske Namestie 14, 811 08 Bratislava, Slovakia; dominikaradosinska@gmail.com; 3 Centre of Experimental Medicine, Slovak Academy of Sciences, Dubravska Cesta 9, 841 04 Bratislava, Slovakia; peter.balis@savba.sk (P.B.); ezgi.dayar@savba.sk (E.D.); iveta.bernatova@savba.sk (I.B.); norbert.vrbjar@savba.sk (N.V.); sona.cacanyova@savba.sk (S.C.); 4 Institute of Experimental Endocrinology, Biomedical Research Center, Slovak Academy of Sciences, Dubravska Cesta 9, 845 05 Bratislava, Slovakia; stefan.zorad@savba.sk

**Keywords:** erythrocytes, SHR, ACE2, MLN-4760, zofenopril, angiotensin, deformability, nitric oxide, Na,K-ATPase, osmotic resistance

## Abstract

Various pathologies (COVID-19 including) are associated with abnormalities in erythrocyte properties. Hypertension represents an unfavorable condition for erythrocyte quality and is the most prevalent risk factor in COVID-19 patients. ACE2 downregulation that is typical of these patients can further deteriorate cardiovascular health; however, its consequences on erythrocyte properties are not known yet. The aim was to investigate the effect of ACE2 inhibition and the potential beneficial effect of zofenopril on erythrocytes in spontaneously hypertensive rats. ACE2 inhibition induced by MLN-4760 (1 mg/kg/day for 2 weeks) led to deterioration of erythrocyte morphology and osmotic resistance, but plasma markers of oxidative stress, erythrocyte deformability, nitric oxide production and Na,K-ATPase activity were not significantly affected. Zofenopril administration (10 mg/kg/day, initiated after 4-day-lasting ACE2 inhibition) resulted in unexpected increase in angiotensin II plasma levels in both control and ACE-inhibited spontaneously hypertensive rats, but in normalization of osmotic resistance in ACE2-inhibited rats. The overall effect of zofenopril on erythrocyte qualities could be evaluated as beneficial.

## 1. Introduction

The most numerous blood elements—red blood cells (RBCs)—are primarily responsible for the transport of respiratory gases between the lungs and tissues. It was shown that RBC properties were deteriorated in COVID-19 patients. Noteworthy, anti-RBC antibodies were detected in 46% of the patients with severe COVID-19, and this was associated with a more frequent need for transfusion as well as development of anemia [1]. In addition, multiple RBC shape abnormalities were observed in these patients. Another case study report also noticed numerous abnormalities in RBC shape as a consequence of their oxidative damage [2]. In the absence of major alterations in standard clinical hematological parameters, RBCs from COVID-19 patients exhibited impaired energy metabolism as well as altered membrane protein and lipid composition [3]. RBCs occupy approximately 40–45% of blood volume. Thus, their properties have significant impact on rheological behavior of blood and quality of circulation. RBCs are able to modify their shape depending on external forces. This ability, also called RBC deformability, regulates erythrocyte rheology, life span, as well as an efficacy of oxygen transport. Among others, the properties of RBC membrane were identified as determinants of RBC deformability [4]. Reported abnormalities in the RBC shape could be responsible for worsening hemorheology in COVID-19 patients. RBC deformability was found lowered in these patients in comparison with septic patients and healthy controls [5]. It was also discussed that the comorbidities responsible for the severity of COVID-19 are actually those associated with the impairment of RBC deformability [6]. Multiple cardiovascular risk factors were identified as related to impairment of RBC deformability [7] as well as adverse outcomes of COVID-19 [8]. Hypertension is the most prevalent risk factor in COVID-19 patients [9]. Inflammation is an integral part in the hypertension and its etiopathogenesis. Matrix metalloproteinase 9 (MMP-9) represents a proteolytic enzyme with multiple functions that include an activation of TNF-α, IL-1β, and TGF-β. MMP-9 is involved in the pathogenesis of numerous diseases such as heart failure and hypertension [10,11]. A relationship between the renin–angiotensin system (RAS), MMP-9 and its inhibitors was established [11]. The inhibition of angiotensin-converting enzyme by enalapril decreased MMP-9 activity in hypertensive patients [12]. It was also shown that the plasma levels of MMP-9 correlated positively with the risk of death in COVID-19 patients [13].

It is generally established that SARS-CoV-2 enters cells via the receptor for angiotensin-converting enzyme 2 (ACE2). The consequent internalization and shedding of ACE2-SARS-CoV-2 complex leads to lower availability of ACE2 that could further deteriorate the cardiovascular health of COVID-19 patients [14]. Despite current guidelines that do not recommend interrupting the treatment affecting RAS, the usage of such a treatment is still under discussion [15]. Several studies suggested that ACE inhibitors (ACEIs) with a sulfhydryl group in the structure, e.g., zofenopril, provide an additional beneficial effect that is additive in comparison with other ACEIs. Zofenopril confers high antioxidant, anti-ischemic and cardioprotective properties that differentiate it from other ACEIs [16]. Furthermore, zofenopril increases hydrogen sulfide (H_2_S) bioavailability [17]. RBCs play an important role in H_2_S metabolism and homeostasis [18] and vice versa, H_2_S modulates RBC properties, e.g., improves their deformability in its optimal concentration [19].

In the present study, we simulated the inhibition of ACE2-mediated signaling induced by SARS-Cov-2 by the usage of highly selective and potent ACE2 inhibitor MLN-4760 (MLN). In this pharmacological model, we intended to examine the extent of MLN-induced RBC damage as well as the mechanisms underlying the action of zofenopril, as perspective pharmacological tool for the treatment of cardiovascular complications associated with COVID-19. Although the presence of ACE2 receptors has not been observed in RBCs, systemic ACE2 inhibition leads to a lowered ability of the organism to metabolize Ang II to Ang 1-7 and prolonged actions of Ang II. The hypothesis of this study was that subsequent consequences of ACE2 inhibition, e.g., the stimulation of inflammation and increase in oxidative stress [13], can affect these blood elements. An induced RBC damage may contrariwise worsen the health status of the organism in the condition of ACE2 inhibition. As an animal model, we used spontaneously hypertensive rats (SHRs) that represent a model of human essential hypertension. SHRs are characterized by increased systolic blood pressure, mean arterial pressure, cardiac and vascular wall hypertrophy, elevated systemic resistance, but unchanged cardiac output [20,21,22]. Remodeling of resistance arteries originating from the difference between the cellular and non-cellular component of the arterial wall affects vasoconstrictor and vasodilator properties of arteries. In addition, dysregulation of several enzymes, in particular catalase, results in oxidative stress that could contribute to the endothelial dysfunction in SHRs [23]. Moreover, Kodavanti et al. [24] demonstrated that SHRs have underlying lung abnormalities: hemorrhage, inflammation, increased vascular leakage, oxidative burden, and associated higher cytokine mRNA expression, including IL-6. In this respect, the proposed model meets the criteria for cardiovascular complications as well as the features of lung injury observed during COVID-19. The first aim of present study was to investigate whether MLN-induced inhibition of ACE2 is detrimental for RBC condition in hypertensive individuals. Consequently, the second goal was to investigate the potential beneficial effect of zofenopril on RBC properties in SHRs after the inhibition of ACE2.

## 2. Materials and Methods

### 2.1. Experimental Model

An animal model of primary hypertension—SHRs—was used in this study. Experimental animals were imported from the breeding facility at the Department of Toxicology and Laboratory Animal Breeding, Centre of Experimental Medicine, Slovak Academy of Sciences, Dobra Voda, Slovak Republic, and were housed in the certified facility at Centre of Experimental Medicine, Institute of Normal and Pathological Physiology, Slovak Academy of Sciences, Bratislava, Slovak Republic. They were kept at two per cage in an air-conditioned room with a 12:12 h light–dark cycle at humidity 45–65% and temperature 22–24 °C. Rats had free access to drinking water and pelleted diet Altromin formula 1324, variant P (Altromin Spezialfutter, Lage, Germany). Animals entered the experiment at the age of 16 weeks and were divided into four groups: control (C; n = 12), control zofenopril-treated (CZ; n = 7), MLN-4760-treated (M; n = 12), and MLN-4760 and zofenopril-treated group (MZ; n = 12).

Specific ACE2 inhibitor MLN-4760 (MLN) (MedChemExpress, Monmouth Junction, NJ, USA) was administered by means of Alzet^®^ mini-osmotic pumps, model 2002, pumping rate 0.5 μL/hour for 14 days (DurectTM, Cupertino, CA, USA) at the dose of 1 mg/kg/day diluted in 10% dimethyl sulfoxide (DMSO) in isotonic saline (sodium chloride 0.9% Braun Intravenous solution for infusion; 308 mOsm/L; 250 mL; B. Braun Meslungen AG, Melsungen, Germany) in M and MZ groups. In C and CZ groups, mini-osmotic pumps were filled with vehicle, i.e., 10% DMSO in isotonic saline. All pumps were implanted subcutaneously on the dorsum of rats under isoflurane (2.5–3%) anesthesia. After implantation of the pumps, the rats were placed in a clean cage and kept in a quiet and warm place until completely recovered from anesthesia.

Systolic blood pressure and body weight were measured 3 days before the beginning of the experiment (starting value) and 1 day before the end of the experiment (end value). The difference between the values was calculated (Δ BP = value at the end minus starting value).

**Figure 1:**
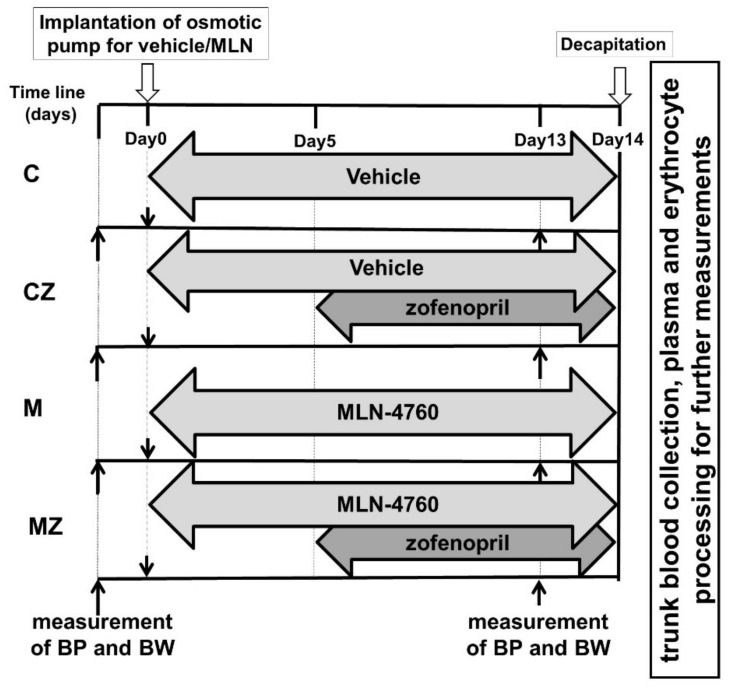
Experimental design. Abbreviations: C—control group, CZ—zofenopril-treated group, M—MLN-treated (MLN-4760, ACE2 inhibitor) group, MZ—zofenopril and MLN-treated group, BP—blood pressure, BW—body weight.

Zofenopril calcium (AdooQ BIOSCIENCE LLC, Irvine, CA, USA) was administered (mixed with food) at the dose of 10 mg/kg/day in CZ and MZ groups. Since the aim was to monitor its effect on an organism that had been exposed to ACE2 inhibition for some time, zofenopril treatment started on day 5 of the MLN administration and lasted till the end of the experiment. On day 14, the rats were sacrificed by decapitation after brief CO_2_ anesthesia. Trunk blood was collected into heparinized tubes (140 UI/5 mL). The hematocrit value, mean cell volume (MCV) and red cell distribution width (RDW-SD) were determined by blood analyzer Sysmex F-820 (Sysmex Corp, Tokyo, Japan). The overall experiment design is presented in Figure 1.

Experimental procedures were approved by the Ethics Committee of the Centre of Experimental Medicine, Slovak Academy of Sciences and by the State Veterinary and Food Administration of the Slovak Republic, with agreement according to European Union Directive 2010/63/EU.

### 2.2. RAS Peptide Concentrations

The equilibrium levels of angiotensin (1-10) (Ang I), angiotensin (1-8) (Ang II), angiotensin (1–7) and angiotensin (1–5) in prestored human frozen heparinized plasma samples were quantified by liquid chromatography mass spectrometry/mass-spectroscopy (LC-MS/MS) in Attoquant Diagnostics (Vienna, Austria) using previously validated methods [25]. The principle of this determination is that the concentrations of different angiotensins are established by equilibrium between their formation and their degradation rates. Ex vivo equilibrium analysis operates without blocking any angiotensin producing or degrading enzymes and takes all soluble factors of angiotensin formation and degradation into account, reflecting the in vivo angiotensin levels and the state of soluble RAS activity. ACE activity marker was calculated based on the angiotensin levels: Ang II/Ang I, since this ratio served as a valid surrogate marker for circulating ACE activity [25]. Alternative RAS activity was calculated as ratio of (Ang1-7 + Ang1-5)/(Ang I + Ang II + Ang1-7 + Ang1-5).

### 2.3. Parameters of Antioxidant Status and Oxidative Stress in Blood Plasma and Hemolyzed RBCs

Markers of oxidative stress, antioxidant status, carbonyl stress, protein oxidation, and lipid peroxidation were determined in plasma samples, according to Kollarova et al. [26]. Spectrophotometric and fluorescent analyses were performed using a TecanSafire II Instrument (Grödig, Austria) and all chemicals used were purchased from the company Sigma-Aldrich (Steinheim, Germany).

The ratio of reduced and oxidized glutathione (GSH/GSSG) was determined as a general marker of oxidative stress. Determination of GSH—10 µL of samples and standards (L-glutathione reduced) was mixed with 10 µL of the ophthaldehyde solution (1 mg/mL) and 180 µL of the phosphate buffer solution (100 mmol/L and 2.5 mM EDTA-Na_2_). Mixtures were incubated and gently vortexed at room temperature (RT) for 15 min. Fluorescence was measured at wavelength λ_ex_ = 350 nm and λ_em_ = 460 nm. Determination of GSSG—25 µL of the samples and standards (glutathione oxidized) was mixed with 10 µL of N-ethylmaleimide (5 mg/mL), vortexed and incubated at RT for 40 min. From the mixture, 10 µL was transferred into the new dark microplate and mixed with 10 µL of the ophthaldehyde solution and 180 µL of NaOH (0.1 mmol/L). Microplates were vortexed and incubated at RT for 15 min and fluorescence was measured at λ_ex_ = 350 nm and λ_em_ = 460 nm.

Ferric reducing antioxidant power (FRAP) was analyzed as the marker of antioxidant status of the samples—200 μL of fresh FRAP reagent (mixture of acetate buffer with pH 3.6, tripyridyl-s-triazine, FeCl_3_*6H_2_O and distilled water) warmed at 37 °C was pipetted into a microplate. Absorbance of FRAP reagent was measured as a blank at λ = 593 nm. Twenty µL of samples and standards (100 mmol/L FeSO_4_*7H_2_O) were added and absorbance was measured after 4 min of gentle vortexing at λ = 593 nm.

Total antioxidant capacity (TAC)—20 μL of samples and standards (mixture of trolox, dimetylsulfoxid and distilled water) was mixed with 200 μL of acetate buffer (pH = 5.8). The absorbance was measured at λ = 660 nm as blank. Twenty μL of ABTS solution (2.2′-azino-bis(3-ethylbenzothiazoline-6-sulfonic acid) with acetate buffer) was added. Absorbance was measured after 5 min of gentle mixing at λ = 660 nm.

Carbonyl stress was measured based on concentration of advanced glycation end products (AGEs)—20 µL of samples was mixed with 180 µL of phosphate-buffered saline in the dark microplate and autofluorescence of the samples was measured at λ_ex_ = 370 nm and λ_em_ = 440 nm. Standards (AGE-BSA) were added and mixed with 180 µL of phosphate-buffered saline. Fluorescence was measured at λ_ex_ = 370 nm and λ_em_ = 440 nm.

Fructosamine was measured as the marker of advanced glycation—20 µL of samples and standards (16 mmol/L 1-deoxy-morpholino-D-fructose) was mixed with 100 µL of 0.25 mmol/L nitro blue tetrazolium (0.1 mmol/L sodium carbonate buffer, pH 10.35 and 1 mmol/L nitro blue tetrazolium). Microplates were gently vortexed and incubated at 37 °C for 15 min, and absorbance was measured at λ = 530 nm.

Advanced oxidation protein products (AOPP) reflect protein oxidation—200 µL of diluted samples and standards (mixture of chloramine-T mixed with potassium iodide) was mixed with 20 µL of glacial acetic acid. Absorbance was measured after 2 min of gentle vortexing at λ = 340 nm.

Lipid peroxidation was measured based on thiobarbituric reactive substances (TBARS)—20 µL of samples and standards (1,1,3,3-tetraethoxypropane) was mixed with 30 µL of distilled water, 20 µL of 0.67% thiobarbituric acid and 20 µL of glacial acetic acid. The microplates were gently vortexed and incubated at 95 °C for 45 min. Afterward, microplates were left to cool to RT. Samples were mixed with 100 µL of n-butanol and centrifuged (10 min, 4 °C, 2000 *g*). The upper phase was transferred into the new dark microplate and fluorescence was measured at λ_ex_ = 515 nm and λ_em_ = 553 nm.

Selected markers of antioxidant status—GSH/GSSG ratio and FRAP were estimated in hemolyzed RBCs.

### 2.4. Measurement of Plasma H_2_S Concentration

Plasma H_2_S concentration was measured via methylene blue assay as described elsewhere [27,28]. Briefly, 75 μL of plasma was mixed with 0.1 mol/L potassium phosphate buffer (325 μL) and was added to a reaction mixture (total volume 500 μL) containing pyridoxal-5-phosphate and L-cysteine to measure H_2_S generation. The reaction was carried out at 37 °C for 30 min. After the incubation, the samples were mixed with trichloroacetic acid (10%, 250 mL), zinc acetate (1%, 250 mL), N, N-dimethyl-p-phenylenediamine sulphate (20 mmol/L, 133 mL) in 7.2 mol/L HCl, and FeCl_3_ (30 mmol/L, 133 mL) in 1.2 mol/L HCl. The mixture was incubated for 10 min at RT and subsequently centrifuged at 12,000× *g* for 2 min. The absorbance of supernatant was measured at λ = 650 nm. All standards and samples were assayed in duplicate. H_2_S was calculated against a calibration curve of Na_2_S (3.9–250 mmol/L). Plasma H_2_S concentration is given in μmol/L.

### 2.5. Matrix Metalloproteinase 9 Activities

Zymography is a semi-quantitative method for determining the activity of matrix metalloproteinases. The detailed method was described elsewhere [26]. The activities of circulating MMP-9 in plasma samples were detected by gelatin zymography in sodium dodecyl sulfate polyacrylamide gel (10%) with incorporated gelatin as a substrate (2 mg/mL). The method involves the electrophoretic separation of proteins in samples under denaturing conditions in a polyacrylamide gel. Briefly, after electrophoresis, the gels were washed twice with 50 mmol/L TRIS (tris (hydroxymethyl) aminomethane, pH 7.4), containing 2.5% Triton X-100. After washing, gels were incubated overnight at 37 °C in a substrate buffer containing 50 mmol/L TRIS (pH 7.4), 10 mmol/L CaCl2, and 1.25% Triton X-100. Afterward, the gels were stained with 1% Coomassie Brilliant Blue G-250 for 4 h and destained with 40% methanol and 10% acetic acid solution. The revealed clear white bands on a blue background represent the MMP-9 gelatinolytic activities. The intensity of these bands was assessed using ImageJ program (NIH, Bethesda, MD, USA). A limitation of this method is the limited count of samples processed in one gel. Thus, the data were separately evaluated for each pair of groups using an unpaired *t* test.

### 2.6. Blood Processing and RBC Isolation

Whole blood was centrifuged (850 g, 10 min, 4 °C). The separated plasma was stored at −80 °C till further analyses. The buffy coat and upper 10% of RBCs were discarded. The remaining RBCs were washed three times in saline. The part of washed RBCs (250 μL) was hemolyzed (final dilution 1:19, *v*:*v*) using cold distilled H_2_O and stored at −80 °C. Changes in RBC morphology were observed by light microscopy.

### 2.7. RBC Deformability

A filtration method was used to determine RBC deformability as in the previous study [29]. RBCs were suspended in manufacturer-formulated Cellpack solution (diluent for Sysmex blood analyzer, 1:1000, *v*:*v*, Sysmex, Slovakia) and centrifuged at 175× *g* for 5 min through membrane filters with 5 μm pores (Ultrafree-MC SV Centrifugal Filter; Merck Millipore Ltd., Tullagreen Carrigtwohill, Ireland). RBC deformability was calculated as the ratio RBCs that passed though the membrane filter and RBC count before centrifugation.

### 2.8. RBC Nitric Oxide Production

In order to visualize RBC-produced nitric oxide (NO), a fluorescent probe 4,5-diaminofluorescein diacetate (DAF-2 DA) was used similarly as described previously [29]. Washed RBCs were diluted 1:19 (*v*:*v*) in phosphate-buffered saline (PBS) (in mmol/L: KCl 2.68, KH_2_PO_4_ 1.76, NaCl 136.89, Na_2_HPO_4_ 10.14, pH 7.4) and treated with DAF-2 DA (25 μmol/L, Abcam, Cambridge, UK). Each sample was incubated for 10 min in the dark at 37 °C. Subsequently, blood smears were prepared. Using filters for fluorescein isothiocyanate (λ_ex_ = 465–495 nm, λ_em_ = 515–555 nm) and fluorescence microscope (Axio Imager M2, Zeiss, Jena, Germany), RBC-related fluorescence signal was captured. Its intensity was quantified using ZEN 3.3 Blue (Carl Zeiss Microscopy GmbH, Germany) and ImageJ 1.53e software (National Institutes of Health, Bethesda, MD, USA). Fluorescence intensity is presented as integrated density of a single RBC.

### 2.9. RBC Free Radical Measurement

The method was modified according to [30]. 2,7-dichlorofluorescin diacetate (DCF) was used as a fluorescent indicator of free radical production by RBCs. Washed RBCs were diluted 1:19 (*v*:*v*) in PBS and treated with DCF (50 μmol/L, Sigma-Aldrich, St. Louis, MO, USA) at 37 °C for 30 min in the dark. Afterward, the sample was briefly centrifuged, and RBCs were resuspended in PBS and prepared for microscopy. The same microscope and filters were used as in DAF-fluorometry. The method of fluorescence quantification was also the same.

### 2.10. Na,K-ATPase Kinetics

The enzyme kinetics of Na, K-ATPase were determined in isolated RBC membranes as described previously [31]. Washed RBCs were homogenized in a 50 mmol/L TRIS and subsequently centrifuged at 13,000× *g* for 30 min at 4 °C. Afterward, the supernatant was discarded, and the sample was homogenized again and centrifuged repeatedly in 30, 20 and 10 mmol/L TRIS to eliminate residual hemoglobin. To determine protein content in each sample, Lowry’s method was used (using bovine serum albumin as a standard). Na,K-ATPase activities were measured in Na^+^ concentrations ranging from 2 to 100 mmol/L (2, 4, 6, 8, 10, 20, 50 and 100). Incubation medium (in mmol/L: MgCl_2_ 4, KCl 10, NaCl 2-100, TRIS 50; pH 7.4) with 50 µg of RBC membrane proteins was preincubated for 20 min at 37 °C, prior to addition of ATP (final concentration 8 mmol/L, Sigma-Aldrich, St. Louis, MO, USA) that was added to start the chemical reaction. After 20 min, it was stopped by adding 0.3 mL of 12% trichloroacetic acid. Inorganic phosphate formed by ATP hydrolysis was determined spectrophotometrically at λ = 700 nm according to Taussky and Shorr [32]. Measured data were used to create kinetic curves, as well as to calculate kinetic parameters of Na,K-ATPase: V_max_—maximum velocity of enzyme reaction and K_Na_—concentration of Na^+^ required for half-maximal activation of the enzyme.

### 2.11. Determination of RBC Osmotic Resistance

RBC osmotic resistance was determined similarly as previously [33]. A set of NaCl solutions (Sigma-Aldrich, St. Louis, MO, USA) in the concentration range from 0% (distilled water) to 0.9% (in % of NaCl: 0, 0.3, 0.4, 0.425, 0.45, 0.5, 0.7, 0.9) was prepared. 10 μL of whole blood was mixed with 1 mL of each NaCl solution. After 30 min incubation at RT, all samples were centrifuged (1200× *g*, 5 min). The extent of hemolysis was measured in supernatants spectrophotometrically at λ = 540 nm. The absorbance of blood samples suspended in 0.9% NaCl was considered as non-hemolytic, and the samples mixed with distilled water were used as equivalent to 100% hemolysis. Afterward, the concentration of NaCl resulting in 50% hemolysis (IC_50_) was calculated. An increase in IC_50_ corresponds to a deterioration in RBC osmotic resistance.

### 2.12. Statistical Analyses

Outliers were detected using the Grubbs’ test and removed from further analyses. Normality of data was analyzed by D’Agostino–Pearson test. Data are presented as means ± standard deviations or as medians with interquartile ranges, where appropriate. Statistical significance was analyzed by two-way ANOVA with main factors: ACE2 inhibition and zofenopril treatment, followed by the Sidak’s multiple comparison test. In case of nonparametric data, we worked with log-transformed data to induce the normal distribution necessary for ANOVA analysis. Differences were considered as significant at *p* < 0.05. Software GraphPad Prism 8.2.1 (GraphPad Software, San Diego, CA, USA) was used for data analysis.

## 3. Results

### 3.1. Basic Biometric Parameters

Regarding blood pressure, statistical analysis did not reveal differences in the comparison of absolute values (in mmHg: 160.8 ± 24.4—start, 170.1 ± 27.93—end in C; 161.6 ± 6.8—start, 156.2 ± 13.2—end in CZ; 162.6 ± 23.5—start, 174.2 ± 30.6—end in M; 159.4 ± 23.3—start, 168.3 ± 18.6—end in MZ group). However, significant differences were found for Δ BP values. The measured values, the significance for the factors as well as for their interaction and the differences between the groups for each parameter are presented in Table 1.

### 3.2. Angiotensin Peptide Concentration in Blood Plasma

**Figure 2:**
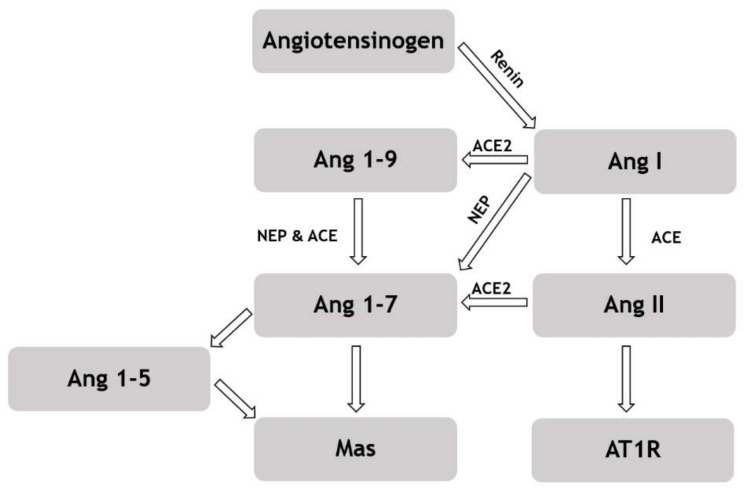
Scheme of the renin-angiotensin system pathways. Abbreviations: Ang—angiotensin, ACE—angiotensin converting enzyme, NEP—neutral endopeptidase—neprilysin, Mas—Mas receptor, AT1R—Angiotensin II type I receptor (modified according to [34]).

Two-way ANOVA revealed zofenopril as a significant factor for concentration of following angiotensins: Ang I, Ang II, Ang 1-5 and Ang 1-7 (for each *p* < 0.0001). For each angiotensin, zofenopril treatment increased its plasma concentration in control rats as well as for ACE2-inhibited rats (for each *p* < 0.01). For Ang II, ACE2 inhibition was a significant factor as well (*p* < 0.05). MLN administration led to the increase in its plasma concentration. Plasma levels of the measured angiotensins as well as the derived parameters (PRA, ACE and ALT) with significances are presented in Table 2. Regarding the relations in the RAS, the scheme is presented in Figure 2.

### 3.3. Parameters of Antioxidant Status and Oxidative Stress in Blood Plasma

Significant differences between groups were revealed in the TAC—higher concentration in MZ compared with M group (*p* < 0.05) and in GSH/GSSG ratio—the ratio was significantly higher in M compared with C (*p* < 0.05) and MZ (*p* < 0.01) group. No other significant differences among the group were detected. A summary of the values as well as statistical significance is available in Table 3.

### 3.4. Parameters of Antioxidant Status in Hemolyzed RBCs

Analysis of GSH/GSSG ratio revealed significance for zofenopril factor (*p* = 0.0044; F_(1,35)_ = 9.248). Zofenopril treatment resulted in a decrease in GSH/GSSG in control rats. This ratio was also lower in M in comparison with C group. In the FRAP value, a significance was revealed for zofenopril factor (*p* = 0.0003; F_(1,36)_ = 16.02) as well as the interaction of zofenopril and ACE2 inhibition (*p* = 0.0009; F_(1,36)_ = 13.22). FRAP was higher in M compared with C group and decreased after zofenopril treatment in ACE2 inhibited rats (Table 4).

### 3.5. Plasma H_2_S Concentration

Plasma H_2_S concentrations followed (in µmol/L): 11.89 ± 2.41 in C; 14.27 ± 3.67 in CZ; 20.77 ± 5.47 in M; 20.24 ± 6.72 in MZ group. Two-way ANOVA revealed only ACE2 inhibition as a significant factor (*p* < 0.0001). Experimental animals from M group showed significantly higher plasma H_2_S concentration in comparison with C group (*p* < 0.001).

### 3.6. Activity of MMP-9 in Plasma

MMP-9 activity in plasma was not modified following ACE2 inhibition (*p* = 0.21, C versus M group). Zofenopril administration resulted in an increase in plasma MMP-9 activity in the control (*p* = 0.0003, C versus CZ group), as well as in ACE2-inhibited (*p* = 0.0001, M versus MZ group) SHRs.

### 3.7. Erythrocyte Parameters MCV and RDW-SD

**Figure 3:**
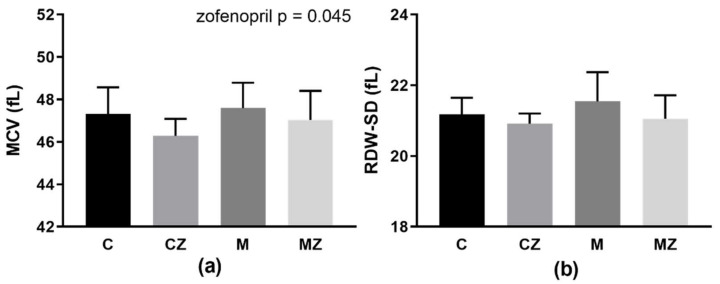
Basic erythrocyte characteristics in experimental groups: MCV (**a**), RDW-SD (**b**). Abbreviations: MCV—mean cell volume, RDW-SD—red cell distribution width, C—control group, CZ—zofenopril-treated group, M—MLN-treated (MLN-4760, ACE2 inhibitor) group, MZ—zofenopril and MLN-treated group. Data are presented as mean ± standard deviations.

Zofenopril treatment significantly lowered MCV (*p* = 0.045; F_(1,39)_ = 4.31) independently of ACE2 inhibition (Figure 3a). In the RDW-SD parameter, there was only tendency to decrease after zofenopril treatment (*p* = 0.061; F_(1,39)_ = 3.73) (Figure 3b).

### 3.8. RBC Deformability and Osmotic Resistance

**Figure 4:**
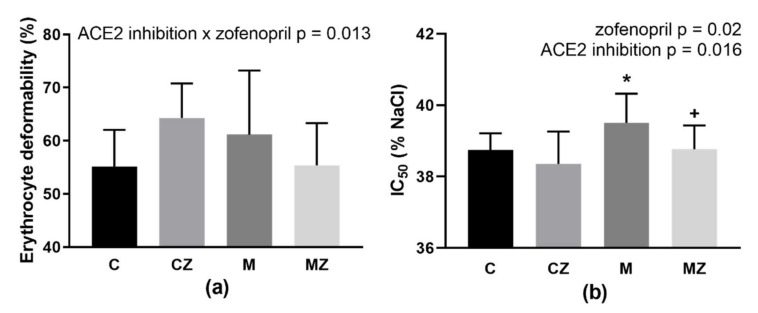
Erythrocyte deformability (**a**) and osmotic resistance (IC_50_) (**b**). Abbreviations: C—control group, CZ—zofenopril-treated group, M—MLN-treated (MLN-4760, ACE2 inhibitor) group, MZ—zofenopril and MLN-treated group. * *p* < 0.05 versus control group, + *p* < 0.05 versus M group. Data are presented as mean ± standard deviations.

Two-way ANOVA revealed a significant ACE2 inhibition × zofenopril interaction (*p* = 0.013; F_(1,37)_ = 6.83) with a non-significant tendency to increased deformability after zofenopril treatment in the control rats (*p* = 0.074) (Figure 4a).

Evaluation of IC_50_ value by two-way ANOVA revealed ACE2 inhibition (*p* = 0.016; F_(1,36)_ = 6.34) as well as zofenopril-treatment (*p* = 0.02; F_(1,36)_ = 5.92) as the significant factors. MLN administration led to increase in IC_50_ (i.e., NaCl concentration at which 50% hemolysis occurred), corresponding to a deterioration in the RBC osmotic resistance. Zofenopril administration improved the ability of RBCs to survive in a hypotonic environment (Figure 4b). The C group had higher osmotic resistance in comparison with M group (*p* = 0.037), while zofenopril treatment led to improvement of osmotic resistance in ACE2-inhibited rats (*p* = 0.037).

### 3.9. Na,K-ATPase Kinetics

**Figure 5:**
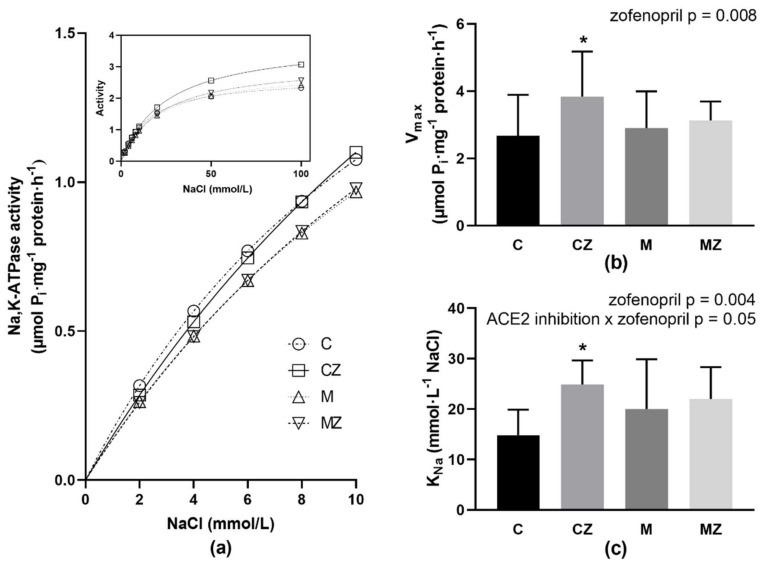
Activation of the Na,K-ATPase in Na^+^ concentrations ranging from 2 to 10 mmol/L; insert—activation of the enzyme in the whole investigated concentration range of NaCl (**a**), kinetic parameters of Na,K-ATPase V_max_ (**b**) and K_Na_ (**c**) in erythrocyte membranes. Abbreviations: C—control group, CZ—zofenopril-treated group, M—MLN-treated (MLN-4760, ACE2 inhibitor) group, MZ—zofenopril and MLN-treated group. V_max_—maximal velocity of reaction. K_Na_—NaCl concentration required for ½ maximal activation of Na,K-ATPase, * *p* < 0.05 versus control group. Data are presented as means ± standard deviations.

Analysis of Na,K-ATPase activation with increasing concentration of NaCl revealed lower activities in RBCs from rats treated with ACE2 inhibitor MLN (M group) as compared with control rats (C group) in the concentration range of NaCl below 10 mmol/L (Figure 5a). Evaluation of the above data by the method of nonlinear regression resulted in similar V_max_ values in C and M groups (Figure 5b). The K_Na_ value showed a tendency to 35% increase (*p* = 0.08) in the M group as compared with the C group, but this change was not statistically significant (Figure 5c).

Two-way ANOVA analysis of V_max_ and K_Na_ parameters revealed zofenopril treatment as significant factor (*p* = 0.008; F_(1,56)_ = 7.57; respectively *p* = 0.004; F_(1,56)_ = 9.12). For K_Na_, also interaction ACE2 inhibition x zofenopril treatment (*p* = 0.05; F_(1,56)_ = 4.02) was significant. Administration of zofenopril induced a biphasic effect on the enzyme activities when comparing the C versus CZ group. In the presence of lower NaCl concentrations—i.e., below 10 mmol/L, the activities of the Na,K-ATPase were similar to controls (CZ versus C). In the presence of higher NaCl concentrations, exceeding 20 mmol/L of NaCl, noticeable stimulation of the enzyme was observed (Figure 5a—insert). Evaluation of observed data revealed significantly increased V_max_ value by 43% (*p* = 0.019) together with increased value of K_Na_ by 67% (*p* = 0.004) in the CZ versus C group (Figure 5b,c).

When investigating the effect of zofenopril on rats treated with MLN, the activation of Na,K-ATPase with increasing NaCl concentration showed similar activities in M and MZ groups in the presence of all applied concentrations of NaCl (Figure 5a). Evaluation of kinetic parameters resulted in similar values of V_max_ and K_Na_ when comparing the M group with the MZ group (Figure 5b,c).

### 3.10. RBC Free Radical and NO Production

**Figure 6:**
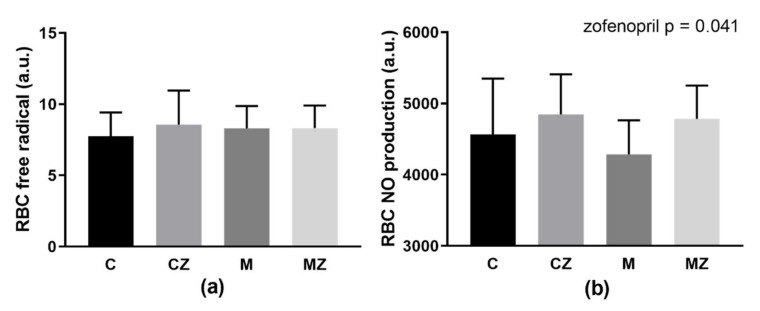
RBC free radical production (**a**) RBC-produced nitric oxide (**b**) Abbreviations: C—control group, CZ—zofenopril-treated group, M—MLN-treated (MLN-4760, ACE2 inhibitor) group, MZ—zofenopril and MLN-treated group, RBC—red blood cell, a.u.—arbitrary units, NO—nitric oxide. Data are presented as means ± standard deviations.

Neither ACE2 inhibition nor zofenopril treatment affected DCF-related fluorescence (Figure 6a). Statistical analysis by two-way ANOVA showed higher NO production by RBCs after zofenopril treatment (*p* = 0.041; F_(1,39)_ = 4.46) independent of ACE2 inhibition (Figure 6b) without differences between groups in multiple comparison test.

### 3.11. Erythrocyte Morphology

**Figure 7:**
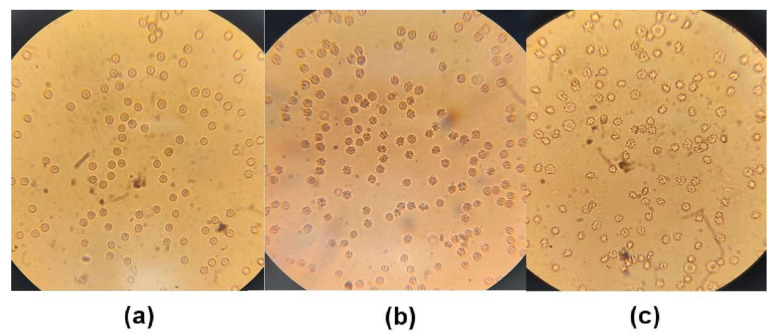
Representative photographs of erythrocyte morphology. Control group (**a**), MLN-4760-treated group (ACE2 inhibition) (**b**), MNL-4760 and zofenopril-treated group (**c**).

In ACE2-inhibited rats (independent of zofenopril), approximately half of the experimental animals had abnormal RBCs, mostly regarding their shape. In ACE2-inhibited animals, membrane wrinkles were more frequent in comparison with control RBCs. Representative photographs are presented in Figure 7.

## 4. Discussion

In RAS cascade, the signaling of one molecule can lead to diverse and even to opposite physiological responses. Ang II production is important in regulation of the sympathetic tone, blood pressure, fluid and ion equilibrium as well as cardiac morphology and function [34,35]. The effect of this molecule—undesirable in its high concentrations—is at least partially antagonized by ACE2-mediated conversion of Ang II to Ang 1-7. The effects of Ang II comprise an increase in blood pressure, sodium and water retention, the promotion of inflammation and cardiac hypertrophy. Contrary, Ang 1-7 promotes vasodilation, inhibits inflammation and offers cardiovascular protection [34,35]. Benefits provided by Ang 1-7 are compromised in SARS-CoV-2 infection due to downregulation of tissue ACE2 receptors. This is considered to be one of the major factors responsible for cardiovascular complications in patients with COVID-19 [14,15,36]. In the present study, two RAS-modulating chemical compounds were administered to experimental animals—two enzyme inhibitors: MLN (ACE2 inhibitor) and zofenopril (ACE inhibitor). Thus, it was crucial to characterize the condition of the experimental animals after both interventions. ACE2 inhibition induced by MLN administration to SHRs resulted in the expected increase in Ang II levels and renin activity, as well as in inhibition of the alternative RAS in blood plasma. However, based on the fact that the absolute concentrations of Ang I and Ang II were approximately 10-fold higher than the concentrations of Ang 1-7 and Ang 1-5, it can be assumed that the key peptides are Ang I and in particular Ang II. In addition, Ang II and Ang 1-7 bind to their corresponding receptors with an affinity of approximately 1 nmol/L (dissociation constant) [35,37]. Therefore, even if the concentration of some angiotensin increases from, e.g., 10 pmol/L to 30 pmol/L, this probably does not have physiological relevance. Thus, mainly changes in Ang II concentrations appear significant. Ang II was paradoxically increased after zofenopril treatment in the present study, the most probably due to 3-4-fold increase in concentration of Ang I that represents the substrate for ACE. It was shown previously that zofenopril administered at the dose 10 mg/kg/day for 3 months decreased ACE activity in plasma by more than 80% [38]. Nevertheless, the remaining activity of ACE could be still sufficient to convert Ang I to Ang II. In the case of sufficient substrate (i.e., Ang I) delivery and in conditions when ACE is not fully saturated, the significant increase in Ang II level may represent the logic consequence. The increase in remaining angiotensins—Ang I, Ang 1-7, Ang 1-5 was expected and confirmed; however, the changes in Ang 1-7 and Ang 1-5 did not seem to reach a level of physiological significance, despite the statistical differences.

The basic anthropometric parameters observed in the present study differ from those that were previously published and documented the lower left ventricular as well as kidney weight and unchanged body weight after zofenopril treatment in SHRs [38]. It may be speculated that, for this disagreement, the starting age of experimental animals might play an important role as the zofenopril dose was the same in both investigations. Another source of above discrepancies may be the different method of data normalization. In the present study, the organ weight was normalized to tibial length, however in the previously published study, they were normalized to body weight. Regarding the changes in parameters of oxidative stress and antioxidant status of experimental animals, ACE2 inhibition surprisingly increased GSH/GSSG ratio together with hydrogen sulfide concentration in blood plasma of SHRs (comparison of C and M groups). It is only a hypothesis at this point that ACE2 inhibition in SHRs may induce some systemic adaptive responses. However, GSH/GSSH ratio was lower and FRAP higher in RBCs taken from M group than from control animals suggesting deterioration of oxidative balance in intracellular compartment following the ACE2 inhibition in this model of genetic hypertension. Ang II is well-known and potent stimulator of inflammatory response in the organism [39]. The higher Ang II concentration can be related, at least partially, to increased plasma MMP-9 activity in both zofenopril-treated groups, as MMP-9 is produced mainly by activated immune cells, mostly macrophages [26].

The proposed experimental study was primarily aimed to identify the consequences of ACE2 inhibition on RBCs of hypertensive rats, and thus to check whether RBC properties could be affected by ACE2 inhibition in an individual suffering from one of the most common cardiovascular risk factors—hypertension. It is important to consider that used experimental animals—SHRs—have already modified RBCs. In these modifications, the deteriorations of deformability and NO production by RBCs together with increase in the osmotic resistance were involved [33]. ACE2 inhibition in SHRs did not result in significant additional damage of RBCs. MLN administration deteriorated morphology of RBCs as well as their osmotic resistance, but the differences between the C and M groups regarding the RBC deformability, NO production, free radical formation, MCV, RDW-SD as well as hematocrit value were not revealed. In an attempt to explain the observed effects of ACE2 inhibition on RBC behavior, the osmotic fragility of RBCs was studied as well. Indeed, the statistically significant deterioration of RBC quality after MLN administration was observed only in osmotic resistance (in addition to their morphology which, however, was the subject of only a semi-quantitative analysis). One may speculate that an adaptive increase in osmotic stability typical for SHRs was abrogated after systemic ACE2 inhibition.

It was repeatedly suggested that cardiovascular functions and cardioprotection is significantly under the control of RBCs [7,40,41,42,43]. RBCs exert series of noncanonical functions, including NO metabolism, control of blood rheology as well as “erythrocrine” signaling by releasing the vasoactive molecules including NO, NO metabolites, and ATP [44]. In cardiovascular pathologies and even in the presence of cardiovascular risk factors, RBC properties are compromised [7]. Noteworthy, the standard therapy of patients suffering from stable angina as well as myocardial infarction could not result in the restoration of the structure and properties of RBCs [45]. Thus, it is necessary to search for new drugs or natural compounds that could normalize RBC abnormalities observed in patients suffering from various cardiovascular diseases. As it was mentioned in the introduction section, zofenopril is ACE inhibitor containing the sulfhydryl group that is offering the antioxidant capacity documented by series of experimental studies and anti-ischemic action documented by clinical trials; for review, see Borghi et al. [46]. The tendency to increase RBC deformability after zofenopril treatment observed only in control SHRs indicates a different response of vehicle- and MLN-treated rats to zofenopril administration, which was at least partially confirmed by the significant interaction of both main factors (ACE2 inhibition and zofenopril). Another parameter of RBCs measuring the broadness of their size distribution—RDW-SD—was suggested to be associated with all-cause mortality and prediction of a poor prognosis in several cardiovascular diseases [47,48]. Borderline significance (*p* = 0.06) for zofenopril treatment regarding this parameter suggests the possible beneficial effect of zofenopril on RBC qualities. Zofenopril was shown to increase NO bioavailability as well as eNOS activity [16]. This knowledge is supported by the observation of the present study; zofenopril increased NO production by RBCs independently of ACE2 inhibition. As RBCs comprise functional eNOS, they can produce NO and this way regulate their own functions as well as functions of vasculature [49]. RBC membrane quality and deformability could be related to alterations in functionality of Na,K-ATPase—the enzyme responsible for ionic and water homeostasis in the cells. The activity of this enzyme is lowered in RBCs of hypertensive individuals, for review see [7]. Thus, the present study also attempted to bring new information concerning the molecular principles of the expected alterations of Na,K-ATPase in RBC membranes after 2 weeks of ACE2 inhibition in SHRs. The investigations using measurements of enzyme activities throughout the applied concentration range of NaCl do not suggest significantly changed Na-binding properties, only the tendency to deterioration after MLN administration as indicated by modification of the K_Na_ value— a comparison of C and M groups. The number of active enzyme molecules remained also unaltered following the ACE2 inhibition, as indicated by similar values of V_max_ in both experimental groups (i.e., C and M groups). The next aim of the present study was oriented to the effect of zofenopril on the Na,K-ATPase properties in SHRs. The enzyme in control rats treated with zofenopril was able to increase its activity in high concentrations of NaCl, where the enzyme from control untreated SHRs was already saturated. This fact may be the most probably explained by increased number of active Na,K-ATPase molecules as indicated by higher value of V_max_ in CZ group as compared with the C group. Zofenopril in rats previously subjected to ACE2 inhibition by MLN did not alter the Na-binding properties or the number of active molecules of Na,K-ATPase in RBCs as indicated by similar values of K_Na_ and V_max_ when comparing the Z and M groups.

Various diseases are associated with abnormalities in RBC properties, especially those associated with a higher degree of oxidative stress [50,51]. RBCs that lack the nucleus and organelles are sensitive to changes in an organism. In addition, due to their short life span (approximately 120 days in humans, 60 days in rats [52]), RBCs can serve as bioindicators of health and disease [51]. One remarkable property of RBCs is their deformability, i.e., the ability to change shape when RBCs are exposed to external forces during their circulation. Less deformable cells are removed from the circulation more quickly as they are trapped by the spleen and subsequently phagocytosed by macrophages. Interestingly, the hematocrit value that reflects RBC removal showed the same changes in this study as the RBC deformability. The significant interaction between both main factors (i.e., ACE2 inhibition and zofenopril administration) suggested improvement of both hematocrit and RBC deformability after zofenopril treatment in control SHRs, but not in ACE2-inhibited ones. Reasonably, a treatment beneficial for hypertensive may be less sufficient for a hypertensive in the condition of ACE2 inhibition when focusing on RBC properties and in particular on their deformability.

## 5. Conclusions

Summarizing the findings of the present study, RBCs are unlikely to be significant victims of ACE2 inhibition in the condition of high blood pressure. Thus, the observed deterioration in RBC quality in SARS-CoV-2 infection is not the consequence of ACE2 downregulation, but most probably the result of oxidative stress. Zofenopril treatment, which surprisingly led to increased Ang II levels in blood plasma of SHRs independent of ACE2 inhibition, resulted in overall beneficial effects on RBC quality.

## Figures and Tables

**Table 1:** Basic biometric parameters.

	C	CZ	M	MZ	Zof.	ACE2i	Inter.
Δ BP (mmHg)	9.28 ± 15.25	−5.36 ± 14.72 ^†^	11.56 ± 18.66	8.83 ± 16.48	x	x	
Δ Body weight (g)	17.65 ± 5.77	9.15 ± 8.67 ^††^	24.85 ± 6.17 ^††^	14.5 ± 8.95 ***	xxxx	xxx	
HW/Tibia (mg/mm)	33 ± 3	31 ± 1	34 ± 2	29 ± 1 ****	xxxx		
LW/Tibia (mg/mm)	344 ± 29	321 ± 16	353 ± 29	327 ± 17 *	xx		
KW/Tibia (mg/mm)	64 ± 3	66 ± 2 ^†^	65 ± 4	63 ± 3			
Hematocrit *(%)*	50.04 ± 3.03	52.46 ± 0.95	51.25 ± 1.48	50.17 ± 2.06			x

Statistical significance: ^†^ *p* < 0.05, ^††^ *p* < 0.01 vs. C; * *p* < 0.05, *** *p* < 0.001, **** *p* < 0.0001 vs. M; x *p* < 0.05, xx *p* < 0.01, xxx *p* < 0.001, xxxx *p* < 0.0001 for factors and their interaction. Abbreviations: Zof—zofenopril administration, ACE2i—ACE2 inhibition, Inter.—interaction between Zof and ACE2i, Δ—difference in the corresponding parameter between the end and the beginning of the experiment, BP—blood pressure, HW—hearth weight, LW—lung weight, KW—kidney weight, C—control group, CZ—zofenopril-treated group, M—MLN-treated (MLN-4760, ACE2 inhibitor) group, MZ—zofenopril and MLN-treated group. Data are presented as mean ± standard deviations.

**Table 2:** Angiotensin concentrations in blood plasma.

	C	CZ	M	MZ	Zof.	ACE2i	Inter.
Ang I (1-10) (pmol/L)	94.5 ± 15.8	372 ± 135.7 ^††††^	105.9 ± 23.7	484.6 ± 112.9 ****	xxxx		
Ang II (1-8) (pmol/L)	161.1 ± 29.3	225.2 ± 33.9 ^††^	186.4 ± 32.2	259.2 ± 32.2 ***	xxxx	x	
Ang 1-7 (pmol/L)	6.6 ± 2.4	16.4 ± 7.0 ^††^	6.4 ± 2.2	19.5 ± 6.3 ****	xxxx		
Ang 1-5 (pmol/L)	20.3 ± 3.1	38.6 ± 9.7 ^††††^	20.9 ± 5.1	46.3 ± 6.9 ****	xxxx		
PRA (pmol/L)	255.6 ± 41.6	597.2 ± 167.1 ^††††^	292.4 ± 53.1	743.8 ± 104 ****	xxxx	x	
ACE	1.7 ± 0.2	0.7 ± 0.1 ^††††^	1.8 ± 0.2	0.6 ± 0.2 ****	xxxx		
ALT	0.096 ± 0.01	0.084 ± 0.005 ^†^	0.084 ± 0.01 ^†^	0.081 ± 0.01	x	x	

Statistical significance: ^†^ *p* < 0.05, ^††^ *p* < 0.01, ^††††^ *p* < 0.0001 vs. C; *** *p* < 0.001, **** *p* < 0.0001 vs. M; x *p* < 0.05, xxxx *p* < 0.0001 for factor. Abbreviations: Ang—angiotensin, PRA—Ang I + Ang II, ACE—Ang II/Ang I, ALT—(Ang 1-7 + Ang 1-5)/(Ang I + Ang II + Ang 1-7 + Ang 1-5). Zof—zofenopril administration, ACE2i—ACE2 inhibition, Inter.—interaction between Zof and ACE2i, C—control group, CZ—zofenopril-treated group, M—MLN-treated (MLN-4760, ACE2 inhibitor) group, MZ—zofenopril and MLN-treated group. Results are presented as mean ± standard deviations.

**Table 3:** The ratio of GSH/GSSG and the concentration of FRAP, TAC, AGEs, FRUC, AOPP and TBARS in the plasma samples.

	C	CZ	M	MZ	Zof.	ACE2i	Inter.
GSH/GSSG	7.05 (5.66; 12.41)	8.74 (7.39; 15.17)	23.94 ^†^ (10.47; 35.46)	5.81 ** (5.14; 16.06)			xx
FRAP (μmol/L)	520 ± 100.7	480 ± 38.8	465 ± 58.1	445 ± 99.9			
TAC (μmol/L)	1.83 ± 0.207	1.86 ± 0.077	1.71 ± 0.194	2.02 ± 0.381 *			
AGEs (g/L)	0.96 ± 0.099	1.00 ± 0.090	0.87 ± 0.125	0.95 ± 0.077		x	
FRUC (mmol/L)	1.34 ± 0.119	1.29 ± 0.127	1.29 ± 0.117	1.23 ± 0.064			
AOPP (μmol/L)	195 ± 63.6	183 ± 54.9	170 ± 57.6	161 ± 48.4			
TBARS (μmol/L)	410 ± 129.0	448 ± 86.2	350 ± 82.7	477 ± 147.3	x		

Statistical significance: ^†^ *p* < 0.05 vs. C; * *p* < 0.05 and ** *p* < 0.01 vs. M; x *p* < 0.05 and xx *p* < 0.01 for factors and their interaction. Abbreviations: C—control group, CZ—zofenopril-treated group, M—MLN-treated (MLN-4760, ACE2 inhibitor) group, MZ—zofenopril and MLN-treated group, GSH—reduced glutathione, GSSG—oxidized glutathione, FRAP—ferric reducing antioxidant power, TAC—total antioxidant capacity, AGEs—advanced glycation end products, FRUC—fructosamine, AOPP—advanced oxidation protein products, TBARS—thiobarbituric reactive substances, Zof—zofenopril administration, ACE2i—ACE2 inhibition, Inter.—interaction between Zof and ACE2i. Results are presented as median and interquartile range for non-parametric data or as mean ± standard deviation for parametric data.

**Table 4:** The ratio of GSH/GSSG and the concentration of FRAP in hemolysate.

	C	CZ	M	MZ	Zof.	ACE2i	Inter.
GSH/GSSG	0.37 ± 0.222	0.16 ± 0.099 ^†^	0.22 ± 0.1 ^†^	0.15 ± 0.051	xx		
FRAP (mmol/L)	13 ± 1.94	12.8 ± 1.36	15.4 ± 2.26 ^†^	10.8 ± 1.40 ***	xxx		xxx

Statistical significance: ^†^ *p* < 0.05 vs. C; *** *p* < 0.001 vs. M; xx *p* < 0.01 and xxx *p* < 0.001 for factor. Abbreviations: GSH—reduced glutathione, GSSG—oxidized glutathione, FRAP—ferric reducing antioxidant power, C—control group, CZ—zofenopril-treated group, M—MLN-treated (MLN-4760, ACE2 inhibitor) group, MZ—zofenopril and MLN-treated group, Zof—zofenopril administration, ACE2i—ACE2 inhibition, Inter.—interaction between Zof and ACE2i. Results are presented as the mean ± standard deviation.

## Data Availability

The data presented in this study are available in this manuscript.
